# Optimizing Image Quality with High-Resolution, Deep-Learning-Based Diffusion-Weighted Imaging in Breast Cancer Patients at 1.5 T

**DOI:** 10.3390/diagnostics14161742

**Published:** 2024-08-10

**Authors:** Susann-Cathrin Olthof, Elisabeth Weiland, Thomas Benkert, Daniel Wessling, Daniel Leyhr, Saif Afat, Konstantin Nikolaou, Heike Preibsch

**Affiliations:** 1 Department of Diagnostic and Interventional Radiology, University Hospital of Tuebingen, 72076 Tuebingen, Germany; saif.afat@med.uni-tuebingen.de (S.A.); konstantin.nikolaou@med.uni-tuebingen.de (K.N.); heike.preibsch@med.uni-tuebingen.de (H.P.); 2 MR Application Predevelopment, Siemens Healthineers AG, 91052 Erlangen, Germany; elisabeth.weiland@siemens-healthineers.com (E.W.); benkert.thomas@siemens-healthineers.com (T.B.); 3 Department of Neuroradiology, University Hospital of Heidelberg, 69120 Heidelberg, Germany; daniel.wessling@med.uni-heidelberg.de; 4 Faculty of Economics and Social Sciences, Institute of Sports Science & Methods Center, University of Tuebingen, 72074 Tuebingen, Germany; daniel.leyhr@uni-tuebingen.de; 5 Cluster of Excellence iFIT (EXC 2180) “Image Guided and Functionally Instructed Tumor Therapies”, University of Tuebingen, 72074 Tuebingen, Germany

**Keywords:** high-resolution deep-learning DWI, breast MRI at 1.5 T, histological proven breast cancer patients

## Abstract

The objective of this study was to evaluate a high-resolution deep-learning (DL)-based diffusion-weighted imaging (DWI) sequence for breast magnetic resonance imaging (MRI) in comparison to a standard DWI sequence (DWI_Std_) at 1.5 T. It is a prospective study of 38 breast cancer patients, who were scanned with DWI_Std_ and DWI_DL_. Both DWI sequences were scored for image quality, sharpness, artifacts, contrast, noise, and diagnostic confidence with a Likert-scale from 1 (non-diagnostic) to 5 (excellent). The lesion diameter was evaluated on b 800 DWI, apparent diffusion coefficient (ADC), and the second subtraction (SUB) of the contrast-enhanced T1 VIBE. SNR was also calculated. Statistics included correlation analyses and paired *t*-tests. High-resolution DWI_DL_ offered significantly superior image quality, sharpness, noise, contrast, and diagnostic confidence (each *p* < 0.02)). Artifacts were significantly higher in DWI_DL_ by one reader (M = 4.62 vs. 4.36 Likert scale, *p* < 0.01) without affecting the diagnostic confidence. SNR was higher in DWI_DL_ for b 50 and ADC maps (each *p* = 0.07). Acquisition time was reduced by 22% in DWI_DL_. The lesion diameters in DWI b 800_DL_ and _Std_ and ADC_DL_ and _Std_ were respectively 6% lower compared to the 2nd SUB. A DL-based diffusion sequence at 1.5 T in breast MRI offers a higher resolution and a faster acquisition, including only minimally more artefacts without affecting the diagnostic confidence.

## 1. Introduction

Dynamic contrast enhanced (DCE)- magnetic resonance imaging (MRI) is the imaging modality with the highest sensitivity for the detection of breast cancer [1]. However, it is an expensive, time-consuming examination, requiring the application of intravenous gadolinium. Linear gadolinium-based contrast agents are also known to cause depositions in the dentate nuclei and globus pallidus of the brain, in the case of repeated intravenous (i.v.) administration; however, without any associated clinical symptoms [2].

Examination time might be reduced through the application of abbreviated multiparametric breast MRI protocols [3] or via a gadolinium-free breast MRI technique, e.g., based on diffusion-weighted imaging (DWI) for tumour detection [4].

DWI plays a crucial role and is routinely used in clinical practice for oncological imaging throughout the whole body [5]. High cellularity as caused, e.g., by tumours, leads to a hindered diffusion with consequently reduced apparent diffusion coefficient (ADC) values. As DWI is sensitive to motion artefacts, single-shot echo-planar imaging (ssEPI) offers an opportunity to limit motion artefacts as it is a fast sequence, acquiring all k-space lines during one single excitation. Although, up to now, DWI has been inferior for the detection of breast cancer in comparison to DCE MRI [6], and not yet established as standard procedure in the BI-RADS catalogue, the European Society of Breast Imaging (EUSOBI) published a consensus recommendation to strengthen its application including essential technical acquisition parameters [7].

Deep-learning plays a crucial role in MRI and is applied to various sequences, e.g., for T1-, T2-, proton density (PD)- and diffusion-weighted images at 1.5 and 3 T [8,9,10,11,12,13]. Its potential is often exploited to speed up the scan, but can also be used to improve image quality and resolution [14]. For DWI, research has already been published, such as from Wessling et al. [8], which focused mainly on the faster acquisition time. In a study by Sauer et al. [9], image quality was additionally improved by using DL-based super resolution while maintaining spatial resolution. To improve spatial resolution, other sequence multi shot types are available, e.g., readout segmentation, where first DL concepts are in sight [15]. However, in this paper, the aim was to improve the image quality in a single shot EPI diffusion sequence by increasing the spatial resolution while maintaining a reasonable scan time. Technique-wise, high spatial resolution is achievable by a novel dedicated super resolution DL for dedicated partial Fourier settings. Radiological evaluation was performed in comparison to the clinically used ssEPI DWI (DWI_Std_) regarding image quality and acquisition time in histological proven breast tumour patients at 1.5 T.

## 2. Materials and Methods

### 2.1. Patient Cohort

**Figure 1:**
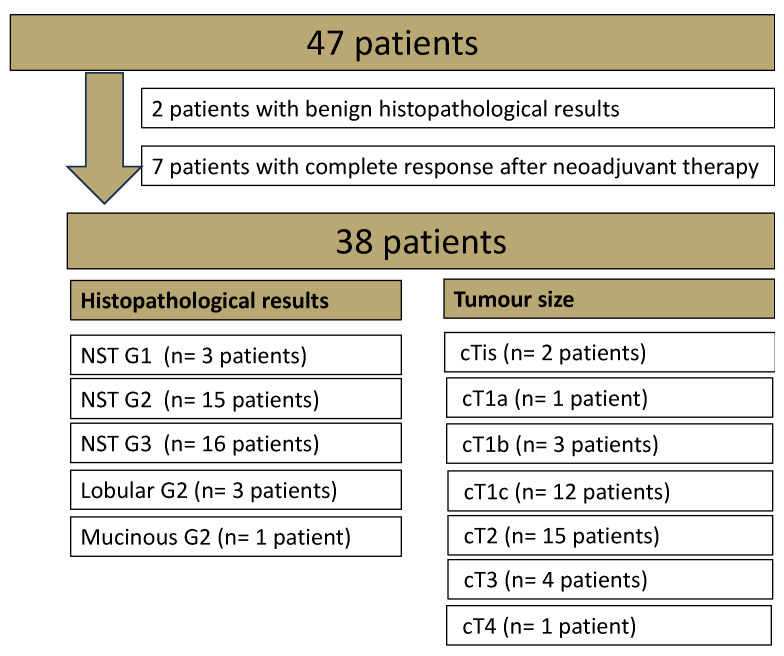
Overview of the histopathological results of the whole study cohort examined in breast magnetic resonance imaging (MRI). Non-special type (NST).

This unicenter, prospective study was approved by the Institutional Review Board of our hospital (055/201BO2). Only patients with signed informed consent were included. The inclusion criteria were histologically proven breast cancer in pre-operative patients without any prior breast cancer, who underwent a breast MRI at 1.5 T with a DWI_Std_ and a research application package DWI_DL_ sequence between March and April 2023 for clinical indications. For details of the histological breast cancer subtypes, see Figure 1.

### 2.2. Image Acquisition

All patients were scanned in a prone position using the same 1.5 T system (MAGNETOM Aera, Siemens Healthineers, Erlangen, Germany) with a dedicated 7-channel bilateral breast coil (Siemens Healthineers, Erlangen, Germany), and received a body weight-adapted dose of i.v. Gadovist (Bayer Healthcare, Berlin, Germany; 0.1 mmol Gadobutrol/kg body weight). Our standard imaging protocol encompassed a T2 fat-suppressed turbo inversion recovery magnitude sequence (T2 TIRM) and non-fat suppressed 3D T1-weighted imaging before and after contrast agent application, as well as a single-shot EPI (ssEPI) acquisition for DWI_Std_ (TE 58 ms; TR 11,700 ms; acceleration factor 2; b values 50 s/mm^2^ and 800 s/mm^2^ with 4 and 16 averages, respectively; no partial Fourier). Additionally, a high-resolution deep-learning-based DWI acquisition (DWI_DL_) was performed (TE 63 ms; TR 12,900 ms; acceleration factor 2; b values 50 s/mm^2^ and 800 s/mm^2^ with 3 and 12 averages, respectively; partial Fourier factor along the phase encoding direction of 6/8). The resolution of DWI_Std_ was 2.2 × 2.2 × 3.0 mm^3^, whereas the DWI_DL_ used a higher in-plane resolution of 0.8 (i) × 0.8 (i) × 3.0 mm^3^. Both DWI_Std_ and DWI_DL_ were obtained during the same clinical scan after contrast media administration. Detailed acquisition parameters are shown in Table 1.

### 2.3. Image Reconstruction

DWI_Std_ images were reconstructed with conventional GRAPPA, while DWI_DL_ images were reconstructed using a research application deep-learning-based reconstruction approach, which contains two different steps. The first uses raw k-space data following the scheme of a variational network [16]. Concretely, 17 unrolled iterations are performed on acquired single-shot EPI data, as well as precalculated coil sensitivity profiles. The first 6 iterations focus on parallel imaging by applying data consistency steps without additional regularization to fill in missing k-space information from PAT undersampling. The remaining 11 iterations focus on denoising by additionally using a regularization term, built via a convolutional neural network with hierarchical down–up architecture. All iterations employ trainable step sizes and Nesterov extrapolation [17]. Training was performed offline in PyTorch, using about 500,000 single-shot DWI images, acquired across different 1.5 T and 3 T clinical MR systems (MAGNETOM, Siemens, Healthineers, Erlangen, Germany) and various body regions. After k-space to image reconstruction, single-shot images were processed in a second step with an image-based super resolution network with pixel shuffle architecture [18]. Here, the goal was to increase sharpness by increasing the matrix size by a factor of two. Furthermore, blurring along the phase-encoding direction due to the applied partial Fourier factor of 6/8 in the acquisition is accounted for by extrapolating the missing 2/8 part of the k-space. By using hard data consistency, only non-measured parts of the k-space were filled in order not to modify the actual image content. To simultaneously achieve the task of super resolution and partial Fourier reconstruction, the network was trained with image pairs consisting of high-resolution images without partial Fourier (ground truth images) and retrospectively downsampled low-resolution images with simulated partial Fourier. Again, training images were acquired in volunteers from different systems and body regions. Both reconstruction steps were trained in a supervised, offline setting. Afterwards, the networks were frozen and integrated into the C++-based reconstruction pipeline at the scanner. After GRAPPA-based reconstruction for DWI_Std_ and deep-learning based reconstruction for DWI_DL_, diffusion specific steps, which included averaging and ADC calculation, were performed identically with the vendor-provided conventional processing steps.

### 2.4. Image Analysis

Two radiologists (H.P with 12 years and S-C.O with 6 years of experience in breast MRI) evaluated first the DWI_Std_, followed by the DWI_DL_ sequences, and ADC_Std_, followed by ADC_DL,_ independently for all patients. Both readers were not blinded for the sequence they evaluated, as the characteristic image impression is obvious for the experienced MR reader. For lesion analysis, only histopathological proven malignant lesions were examined. All included patients were surgical treated inhouse and histopathological specimens were analysed in our local histopathology department (for details see Figure 1). Benign lesions (*n* = 9) were omitted in the analysis. 

Each reader evaluated DWI_Std_, and DWI_DL_ for b values 50 and 800, as well as ADC_Std_ and ADC_DL_ sequences qualitatively and quantitatively in our standard postprocessing software (syngo.via, 9.4, Siemens Healthineers, Erlangen, Germany). 

Qualitative evaluation of the malignant lesion was based on a five-point Likert scale (with 1 for non-diagnostic imaging, 2 for poor, 3 for moderate, 4 for good and 5 for excellent) for image quality, sharpness, artifacts, contrast, noise and diagnostic confidence.

Quantitative analysis included the diameter of the malignant lesion in DWI_Std_ and DWI_DL_ at b 800 and ADC_Std_ and ADC_DL_ compared to our gold standard in the 2nd SUB sequence. For both DWI_Std_ and DWI_DL_, SNR was analysed on both b-values, and ADC values were investigated by applying an oval-shaped two-dimensional ROI of 20 mm^2^ in each breast quadrant. Once a ROI was placed, it was copied to the same region in all sequences. The SNR values were obtained by the quotient of the mean and standard deviation [19].

### 2.5. Statistical Analysis

Statistical analysis was performed using SPSS (version 28, IBM, Chicago, IL, USA). Descriptive statistics were displayed as mean values with standard deviation. Median and IQR values were neglected, as those would not have been informative due to ratings based on a five-point Likert scale. For inferential statistics, the significance level was set to α = 0.05. Inter-reader reliability between the two readers were analysed via Pearson correlations. Paired sample *t*-tests were applied for the analyses of differences in image quality, lesion detection, and acquisition time between DWI_Std_ and DWI_DL_. Beforehand, all outcomes were checked for the assumption of normal distribution. Whenever the assumption could not be confirmed, additional parameter-free tests (i.e., Spearman rank, and Wilcoxon tests) were calculated. These led to the same statistical test decisions and similar effect sizes as the parametric tests carried out. To be consistent in the presentation and comparison of all results, we therefore decided to report only the parametric tests in the results section.

## 3. Results

### 3.1. Subsections

#### 3.1.1. Patients

Of the 47 patients, 2 had histologically proven benign disease (adenosis), and another 7 patients revealed a complete response after systemic therapy (Figure 1). The mean age of the included 38 female patients was 54.5 years (SD 12.35). The mean lesion diameter, measured on the T1-weighted 2nd SUB, was 25.4 mm (SD 16.7).

#### 3.1.2. Qualitative Image Evaluation

##### Qualitative Image Evaluation for DWI

Regarding all analysed imaging parameters (image quality, sharpness, artifacts, image contrast, noise and diagnostic confidence), DWI_DL_ revealed significantly superior results compared to DWI_Std_ at b 50 and b 800 values for both readers (each *p* < 0.02), except for chemical shift artifacts in high-resolution DWI_DL_ in reader 2 (*p* = 0.01, Table 2). Inter-reader reliability was best for image quality in DWI_Std_ (r = 0.74), sharpness in DWI_DL_ (r = 0.78), contrast in DWI_Std_ (r = 0.73), artifacts in DWI_Std_ (r = 0.70) and diagnostic confidence in DWI_Std_ (r = 0.95) and DWI_DL_ (r = 0.91; Table 2).

##### Qualitative Image Evaluation for ADC

**Figure 2:**
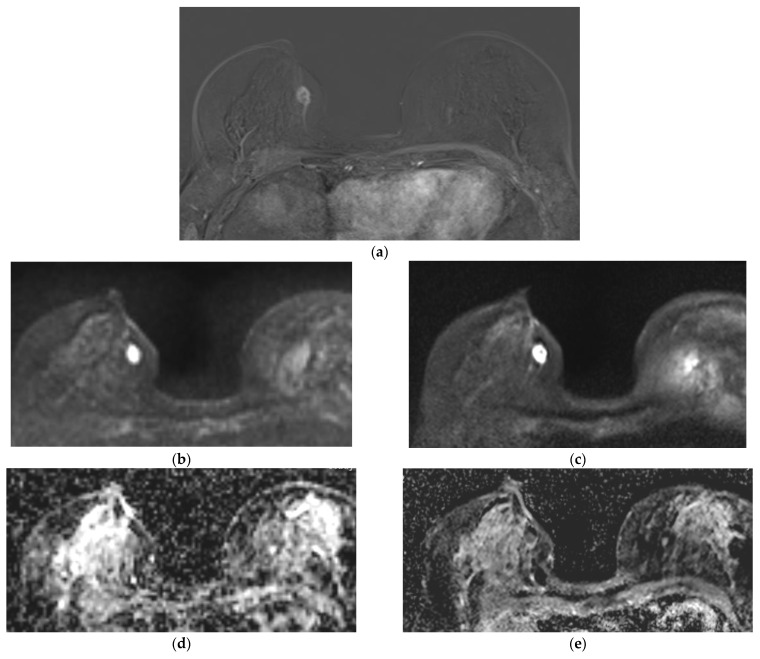
A 68-year-old patient with histologically proven breast carcinoma, non-special type (NST), G2 on the right side. The 2nd subtraction (SUB) is the diagnostic gold-standard (**a**). The lesion in diffusion-weighted imaging (DWI)_Std_ (**b**) was less sharp, compared to DWI_DL_ (**c**). Additionally, visibility for lesion detection was in apparent diffusion coefficient (ADC)_Std_ inferior (**d**) to ADC_DL_ (**e**).

ADC_DL_ was significantly superior to ADC_Std_ for all analysed parameters (image quality, sharpness, artifacts, image contrast, noise and diagnostic confidence) in both readers (each *p* < 0.03 Table 3, Figure 2). Inter-reader reliability was best for diagnostic confidence in ADC_Std_ (r = 0.74) and lowest for image quality in ADC_Std_ (r = 0.37), and artifacts in ADC_Std_ (r = 0.47) and ADC_DL_ (r = 0.30; Table 3).

#### 3.1.3. Quantitative Image Evaluation

##### Lesion Visibility and Diameter

The primary tumour was visible in all analysed sequences (*n* = 38). 

Compared to the gold standard of the 2nd T1w SUB after contrast media application, the lesion diameter was 6.1% lower in DWI_Std_ and DWI_DL_.

**Figure 3:**
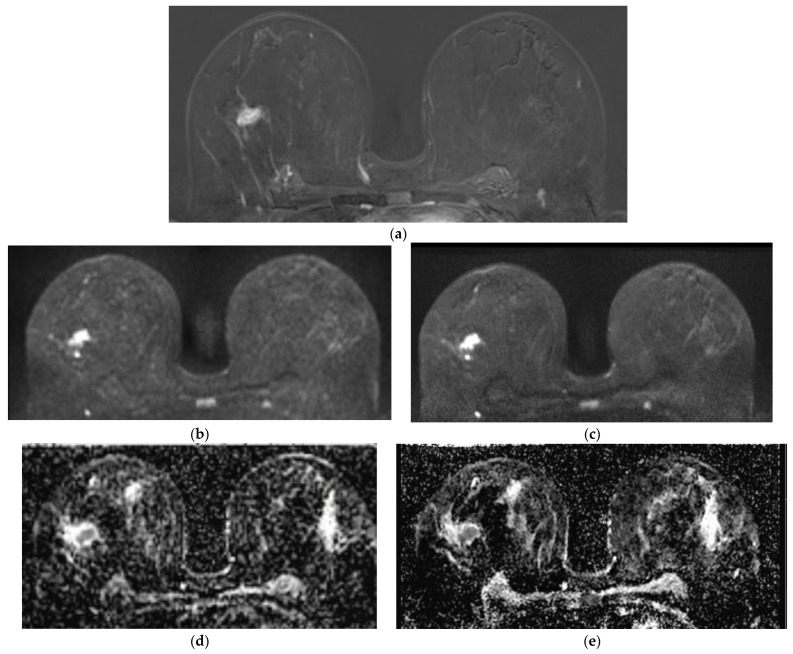
A 54-year-old patient with histologically proven breast carcinoma, NST, G2 on the right side in the 2nd subtraction (**a**). The exact lesion diameter could be determined more clearly in DWI_DL_ (**c**) and ADC_DL_ (**e**) compared to DWI_Std_ (**b**) and ADC_Std_ (**d**).

Regarding the lesion diameter in ADC, 5.2% and 7.2% smaller lesion diameters were measured in ADC_Std_ and ADC_DL_ in comparison to the gold-standard of the 2nd SUB (Table 4, Figure 3). The signal intensities of the lesions revealed significantly higher values for ADC_DL_ compared to ADC_Std_ (*p* = 0.02; Table 5).

##### SNR

SNR in DWI_DL_ was higher than in DWI_Std_ for b 50; however, it was not statistically significant (*p* = 0.07 and 0.06). A comparable SNR was obtained for DWI_DL_ and DWI_Std_ at b 800 values (*p* = 0.92; Table 6).

##### Image Acquisition Time

The acquisition time was 3:49 min for DWI_DL_ compared to 4:54 min for DWI_Std_, offering the patients a 22% shorter examination time.

## 4. Discussion

Comparing high-resolution DWI_DL_ with DWI_Std_, all lesions were visible in both sequences, indicating that DWI_DL_ is a clinically applicable and useful technique at 1.5 T. With a mean size of 25 mm, no lesions were missed in our DWI_DL_. Furthermore, the DWI_DL_ sequence offered a mean scanning time reduction of 65 s in our study.

Breast MR examinations for cancer screening in high-risk patients are steadily increasing, according to the detection of new genetic risk profiles [20]. As these women are commonly young and have to undergo at least a yearly MRI examination, a reduction of examination time and a potential substitute for gadolinium contrast-agents would be a great step in the diagnostic work. Although this issue has been examined for several years with inferior results for DWI compared to dynamic CE breast MR [21], further development of DL sequences in DWI might have the potential to overcome this problem, as our study demonstrates with no missed lesions in high-resolution DWI_DL_. Furthermore, our results indicate that the exact tumor diameter can be measured with the high-resolution DWI_DL_ b 800 and ADC_DL_ compared to the gold-standard of the 2nd SUB, which is crucial for planning the individual therapeutic concept [22].

Regarding the choice of applied b values in breast DWI, our protocol encompassed b 800 values, which is in line with most of the published breast DWI studies for good diagnostic specificity and an acceptable SNR [23].

In general, DL-based techniques have been shown to result in higher SNR values, not only in T1 VIBE, and PD sequences, but also in DWI in musculoskeletal and abdominal imaging [11,12,13]. In our study, SNR for DWI_DL_ compared with DWI_Std_ was higher for b 50 and ADC, however not statistically significant (*p* = 0.073 and 0.069). One potential reason is that DWI_DL_ used an increased matrix size, which in turn reduces overall SNR.

So far, deep-learning-based DWI at 1.5 T has been applied for faster image acquisition while maintaining equal image quality, contrast and diagnostic confidence [8]. With the proposed DWI_DL_ sequence, both superior image quality and a reduction of acquisition time compared to conventional DWI is feasible as the analysed image quality, sharpness, noise, contrast and diagnostic confidence were significantly higher in high-resolution DWI_DL_ compared to DWI_Std_. Thus, this new high-resolution DWI represents a relevant development for DWI_DL_ sequences in breast MRI at 1.5 T and can strengthen the role of DWI in the clinical routine in the staging and high-risk screening population. As a consequence, abbreviated breast MRI for screening in high-risk patients might, in the future, potentially be possible without i.v. CM application in the clinical routine. 

At 3 T, including benign and malignant lesions, the DL DWI sequence resulted in significantly higher contrast, while the SNR and contrast-to-noise values were comparable between DWI _Std_ and DWI _DL_ [10]

While high-resolution DWI_DL_ was rated superior in almost all categories, reader 2 stated increased chemical shift artifacts compared to DWI_Std_; however, without affecting the diagnostic confidence in both readers (M = 4.62 vs. M = 4.36 in Likert scale, *p* 0.01). Given that a higher matrix size and therefore longer echo trains were used for DWI_DL_, this is expected and independent from the DL reconstruction. Employing segmented instead of single-shot readouts might help to reduce chemical shift, which should be investigated in further studies for breast MR at 1.5 T.

The limitations of this study include the small study cohort. However, we provided a patient collective with histologically proven breast cancers. In the future, larger patient cohorts, suffering from the same tumour histology and grading type, should be analysed to gain more information regarding the homogenous data of DL in DWI, potentially enabling cut-off values for benign vs. malignant lesions.

## 5. Conclusions

High resolution DL-based DWI in breast MR at 1.5 T offers superior diagnostic image quality compared to conventional DWI, while reducing the acquisition time by up to 22%. This result strengthens the role of DWI for implementation in clinical diagnostic routines, and might potentially also play a crucial role in the evaluation of gadolinium-free breast examinations. Further studies with larger patient cohorts should be performed for validation of these initial results. Additionally, homogenous cohorts should be analysed independently at 1.5 and 3 T, to gain further knowledge, potentially enabling a differentiation between benign and malignant breast lesions through the identification of a cut-off value for b 50, b 800 and ADC values.

## Figures and Tables

**Table 1:** Details of the protocol parameters of diffusion weighted imaging (DWI)_Std_ and DWI_DL_.

Protocol Parameter	DWI_Std_	DWI_DL_
Resolution	2.2 × 2.2 × 3.0 mm	0.8 (i) × 0.8 (i) × 3.0 mm
Acquisition time (TA)	4:54 min	3:49 min
Repitition time (TR)/ Echo time (TE)	11,700/58 ms	12,900/63 ms
Fat Saturation	Spectral attenuated inversion recovery (SPAIR)	SPAIR
Parallel imaging factor	2	2
b-values (averages)	50 (4)/800 (16) s/mm^2^	50 (3)/800 (12) s/mm^2^
Diffusion mode	3D Diagonal	3D Diagonal
Partial Fourier	None	6/8
Deep Learning (DL)	None	DL reconstruction, DL super resolution

**Table 2:** Visual evaluation of the image quality (IQ), sharpness, noise, contrast, artifacts and diagnostic confidence (DC) for DWI_Std_ and _DL_ in both readers.

	Reader 1			Reader 2			Interreader Reliability (r)	
Image Parameters DWI Sequence	DWI_Std_ Mean (SD)	DWI_DL_ Mean (SD)	*p*-Value	DWI_Std_ Mean (SD)	DWI_DL_ Mean (SD)	*p*-Value	DWI_Std_	DWI_DL_
Overall Image Quality								
IQ ^1^	3.86 (0.58)	4.49 (0.65)	<0.001	3.92 (0.54)	4.70 (0.46)	<0.001	0.746	0.585
Sharpness	3.86 (0.67)	4.68 (0.58)	<0.001	3.78 (0.41)	4.76 (0.49)	<0.001	0.684	0.782
Noise	4.00 (0.68)	4.65 (0.63)	<0.001	4.08 (0.49)	4.78 (0.47)	<0.001	0.403	0.567
Contrast	4.49 (0.76)	4.65 (0.67)	0.010	4.36 (0.68)	4.62 (0.54)	0.010	0.730	0.609
Artifacts	4.16 (0.72)	4.43 (0.76)	0.020	4.46 (0.55)	4.16 (0.60)	0.010	0.702	0.387
DC ^2^	4.49 (0.98)	4.59 (0.89)	0.100	4.59 (0.83)	4.73 (0.60)	0.020	0.955	0.915

^1^ IQ = image quality; ^2^ DC = diagnostic confidence; IQR = interquartile range for values in brackets.

**Table 3:** Visual evaluation of the image quality (IQ), sharpness, noise, contrast, artifacts and diagnostic confidence (DC) for ADC_Std_ and _DL_ in both readers.

	Reader 1			Reader 2			Interreader Reliability (r)	
Image Parameters ADC	ADC_Std_ Mean (SD)	ADCI_DL_ Mean (SD)	*p*-Value	ADC_Std_ Mean (SD)	ADC_DL_ Mean (SD)	*p*-Value	ADC_Std_	ADC_DL_
**Overall Image Quality**								
IQ ^1^	3.41 (0.59)	3.95 (0.91)	<0.001	3.46 (0.50)	4.11 (0.51)	<0.001	0.377	0.486
Sharpness	3.41 (0.68)	4.05 (0.91	<0.001	3.41 (0.59)	4.16 (0.72)	<0.001	0.671	0.615
Noise	3.24 (0.64)	3.62 (0.89)	<0.001	3.24 (0.49)	3.73 (0.69)	<0.001	0.596	0.548
Contrast	3.51 (0.83)	3.76 (0.89)	<0.001	3.86 (0.48)	3.62 (0.54)	0.020	0.522	0.376
Artifacts	3.41 (0.68)	3.81 (0.93)	<0.001	3.78 (0.53)	3.73 (0.69)	0.660	0.474	0.30
DC ^2^	3.62 (1.06)	3.97 (1.04)	<0.001	3.62 (1.01)	3.78 (1.03)	0.030	0.743	0.668

^1^ IQ= image quality; ^2^ DC = diagnostic confidence; ^3^ IQR = interquartile range for values in brackets.

**Table 4:** Lesion diameters in mm measured in DWI b 800_Std_ and _DL_ and ADC_Std_ and _DL._ in comparison to the gold-standard of the 2nd SUB.

Lesion Size in mm	_Std_	_DL_
DWI b 800	24.13 (−4.90%)	23.94 (−5.7%)
ADC (mm^2^/s)	24.00 (−5.20%)	23.48 (−7.2%)
2nd SUB	25.3	

**Table 5:** Overview of quantitative image parameters obtained in DWI_Std_ and _DL_ at b 50 and b 800 values, as well as in ADC maps.

Quantitative Image Parameters (Lesion)	DWI_Std_ Mean (SD ^1^)	DWI_DL_ Mean (SD ^1^)	*p*-Value
ADC (mm^2^/s)	936.22 (262.47)	980.78 (274.00)	0.02

^1^ SD = standard deviation.

**Table 6:** Noise and SNR measured in DWI_Std_ and _DL_, both at b 50 and b 800 values and in ADC maps.

Noise	DWI_Std_ SD ^1^	DWI_DL_ SD ^1^	*p*-Value	SNR ^2^	DWI_Std_ SNR ^2^	DWI_DL_ SNR ^2^	*p*-Value
b 50	96.78	88.61	0.001	b 50	4.17	4.36	0.073
b 800	29.72	27.59	<0.001	b 800	7.27	7.29	0.925

^1^ SD = standard deviation, ^2^ SNR = signal-to-noise-ratio;.

## Data Availability

The data presented in this study are available on request from the corresponding author due to privacy.

## References

[1] Mann R.M., Balleyguier C., Baltzer P.A., Bick U., Colin C., Cornford E., Evans A., Fallenberg E., Forrai G., Fuchsjager M.H. (2015). Breast MRI: EUSOBI recommendations for women’s information. Eur. Radiol..

[2] van der Molen A.J., Quattrocchi C.C., Mallio C.A., Dekkers I.A., European Society of Magnetic Resonance in Medicine, Biology Gadolinium Research, Educational Committee (ESMRMB-GREC) (2024). Ten years of gadolinium retention and deposition: ESMRMB-GREC looks backward and forward. Eur. Radiol..

[3] Kuhl C.K., Schrading S., Strobel K., Schild H.H., Hilgers R.D., Bieling H.B. (2014). Abbreviated breast magnetic resonance imaging (MRI): First postcontrast subtracted images and maximum-intensity projection-a novel approach to breast cancer screening with MRI. J. Clin. Oncol. Off. J. Am. Soc. Clin. Oncol..

[4] Sardanelli F., Carbonaro L.A., Montemezzi S., Cavedon C., Trimboli R.M. (2016). Clinical Breast MR Using MRS or DWI: Who Is the Winner?. Front. Oncol..

[5] Obara M., Kwon J., Yoneyama M., Ueda Y., Cauteren M.V. (2023). Technical Advancements in Abdominal Diffusion-weighted Imaging. Magn. Reson. Med. Sci..

[6] Zhang L., Tang M., Min Z., Lu J., Lei X., Zhang X. (2016). Accuracy of combined dynamic contrast-enhanced magnetic resonance imaging and diffusion-weighted imaging for breast cancer detection: A meta-analysis. Acta Radiol..

[7] Baltzer P., Mann R.M., Iima M., Sigmund E.E., Clauser P., Gilbert F.J., Martincich L., Partridge S.C., Patterson A., Pinker K. (2020). Diffusion-weighted imaging of the breast-a consensus and mission statement from the EUSOBI International Breast Diffusion-Weighted Imaging working group. Eur. Radiol..

[8] Wessling D., Gassenmaier S., Olthof S.C., Benkert T., Weiland E., Afat S., Preibsch H. (2023). Novel deep-learning-based diffusion weighted imaging sequence in 1.5 T breast MRI. Eur. J. Radiol..

[9] Sauer S.T., Christner S.A., Lois A.M., Woznicki P., Curtaz C., Kunz A.S., Weiland E., Benkert T., Bley T.A., Baessler B. (2023). Deep Learning k-Space-to-Image Reconstruction Facilitates High Spatial Resolution and Scan Time Reduction in Diffusion-Weighted Imaging Breast MRI. J. Magn. Reson. Imaging.

[10] Wilpert C., Neubauer C., Rau A., Schneider H., Benkert T., Weiland E., Strecker R., Reisert M., Benndorf M., Weiss J. (2023). Accelerated Diffusion-Weighted Imaging in 3 T Breast MRI Using a Deep Learning Reconstruction Algorithm With Superresolution Processing: A Prospective Comparative Study. Investig. Radiol..

[11] Kiryu S., Akai H., Yasaka K., Tajima T., Kunimatsu A., Yoshioka N., Akahane M., Abe O., Ohtomo K. (2023). Clinical Impact of Deep Learning Reconstruction in MRI. Radiographics.

[12] Chaika M., Afat S., Wessling D., Afat C., Nickel D., Kannengiesser S., Herrmann J., Almansour H., Mannlin S., Othman A.E. (2022). Deep learning-based super-resolution gradient echo imaging of the pancreas: Improvement of image quality and reduction of acquisition time. Diagn. Interv. Imaging.

[13] Herrmann J., Feng Y.S., Gassenmaier S., Grunz J.P., Koerzdoerfer G., Lingg A., Almansour H., Nickel D., Othman A.E., Afat S. (2024). Fast 5-minute shoulder MRI protocol with accelerated TSE-sequences and deep learning image reconstruction for the assessment of shoulder pain at 1.5 and 3 Tesla. Eur. J. Radiol. Open.

[14] Gassenmaier S., Warm V., Nickel D., Weiland E., Herrmann J., Almansour H., Wessling D., Afat S. (2023). Thin-Slice Prostate MRI Enabled by Deep Learning Image Reconstruction. Cancers.

[15] Liu W., Darwish O., Benkert T., Weiland E., Nickel M.D. (2024). Improved Readout-Segmented EPI Using Deep Learning Reconstruction. https://submissions.mirasmart.com/ISMRM2024/Itinerary/Login.aspx.

[16] Hammernik K., Klatzer T., Kobler E., Recht M.P., Sodickson D.K., Pock T., Knoll F. (2018). Learning a variational network for reconstruction of accelerated MRI data. Magn. Reson. Med..

[17] Tao W., Pan Z., Wu G., Tao Q. (2020). The Strength of Nesterov’s Extrapolation in the Individual Convergence of Nonsmooth Optimization. IEEE Trans. Neural Netw. Learn. Syst..

[18] Shi W., Caballero J., Huszár F. Real-time single image and video super-resolution using an efficient sub-pixel convolutional neural net-work. Proceedings of the IEEE Conference on Computer Vision and Pattern Recognition (CVPR).

[19] Song S.E., Woo O.H., Cho K.R., Seo B.K., Son Y.H., Grimm R., Liu W., Moon W.K. (2021). Simultaneous Multislice Readout-Segmented Echo Planar Imaging for Diffusion-Weighted MRI in Patients With Invasive Breast Cancers. J. Magn. Reson. Imaging.

[20] Sheikh A., Hussain S.A., Ghori Q., Naeem N., Fazil A., Giri S., Sathian B., Mainali P., Al Tamimi D.M. (2015). The spectrum of genetic mutations in breast cancer. Asian Pac. J. Cancer Prev..

[21] Partridge S.C., Nissan N., Rahbar H., Kitsch A.E., Sigmund E.E. (2017). Diffusion-weighted breast MRI: Clinical applications and emerging techniques. J. Magn. Reson. Imaging.

[22] Wekking D., Porcu M., De Silva P., Saba L., Scartozzi M., Solinas C. (2023). Breast MRI: Clinical Indications, Recommendations, and Future Applications in Breast Cancer Diagnosis. Curr. Oncol. Rep..

[23] Messina C., Bignone R., Bruno A., Bruno A., Bruno F., Calandri M., Caruso D., Coppolino P., Robertis R., Gentili F. (2020). Diffusion-Weighted Imaging in Oncology: An Update. Cancers.

